# The Trichina Spiralis

**Published:** 1866-08

**Authors:** E. M. Smith

**Affiliations:** Marion, Linn County, Iowa


### CHICAGO MEDICAL JOURNAL.

VOL. XXIII. AUGUST, 1866. NO. 8.

### ORIGINAL CONTRIBUTIONS.

*The Trichina Spiralis. A Family Poisoned by Eating Trichinous Pork.* By E. M. SMITH, M. D., of Marion, Linn County, Iowa.

In view of the great excitement existing at the present time, in Central Iowa, respecting the prevalence of the disease called Trichiniasis, caused by eating raw trichinous pork, I have determined to prepare a correct report of the cases which have given rise to so much interest and inquiry. That the malady exists in this section of the country, is no longer a matter of doubt.

So long as the disease was distant from us, and our principal knowledge of it consisted of unauthenticated newspaper gleanings, and occasionally a brief notice of the subject by the medical journals, we were content to almost ignore the very existence of the trichinal disease, or, at least, to more than half believe that the disease was rather a speculative theory than a practical matter to be encountered by the profession.

On the morning of May 11th, 1866, I was requested to call at the residence of a Mr. Bemis, the messenger remarking that "they were all sick and didn't know what was the matter." The family occupied a comfortable suit of rooms on the second floor over a clothing store, in a sufficiently ventilated building, which is evidently as healthy as most dwellings. I am thus particular as to the rooms, that there may be no false conjecture of oclesis or crowd-poison in the case. The family numbered ten persons: Mr. Bemis, aged 72; Mrs. B., aged 57; two unmarried sons—Henry, aged 23, and Whitfield, aged 20; a son-in-law, Mr. Lansing, with his wife and four children, aged, respectively, 13, 9 and 6, the last being twins. I called as requested, and found six persons lying ill: Mr. B., his wife and son, Henry B., together with three of Mr. Lansing's children—Willie, Ella and Albert. I learned that all six had been taken sick at about the same time, a week previous to my call. The prominent symptoms observable at the time were: more or less diarrhœa; tenderness of the abdomen; tongue thickly coated, contracted and red about the edges; œdema of the face in several of the cases; considerable pneumonic irritation in some; fever of a typhoid character; pulse ranging from 100 to 120 per minute; great thirst and profuse sweating; complaint of soreness and stiffness of the muscles, a difficulty of extending the limbs, wakefulness at night, and excessive exhaustion. Each case exhibited more or less of the symptoms just enumerated, the difference being merely in intensity.

It seemed to me something remarkable that so many persons should all be taken ill at about the same time, in the same family, and with the same general range of symptoms, while no sickness of like character was prevailing in the community. Making a careful examination of each patient, I informed the family that I was fearful that it would prove to be the trichinal disease; still I was not positive, never having before seen a case; and further, having made diligent inquiry in regard to the previous diet of the family, I could elicit no facts which would lead me to believe that any raw pork or other meat even had been eaten. But I was not satisfied, and so concluded to watch my patients awhile before giving a positive diagnosis, and to prescribe such palliative remedies as were indicated, my opinion being divided between trichiniasis and typhoid fever.

On the 14th of May, three other members of the family (Whitfield B., Mrs. Lansing and her youngest daughter) began to complain of like symptoms with the first six; and so impressed was I with the idea of trichiniasis, that I ordered each an active cathartic. They and one other case have recovered.

The œdematous condition of the face disappeared about the fourth or fifth day after its appearance, and immediately swelling of the ankle joints, with some soreness, supervened; while two of the cases showed decided anasarca of the lower extremities, which continued until death. During the second week of my attendance but very little change was observable in a majority of the cases, excepting that three of the milder cases began to convalesce, and a gradual increase of the pneumonic difficulty occurred in Mrs. B. and Henry B. In the latter case it became so severe as to require active treatment for several days; respirations, 48 per minute, with copious brick-dust colored expectoration. I believe that in this case the pneumonic affection was much aggravated by sitting in a draft before an open window while perspiring freely. At this time several of the patients were somewhat delirious at night, but had shown little or no indications of coma.

On May 30th, Willie Lansing, seemingly doing well, suddenly grew worse, and in the evening I called in consultation my friends, Drs. Bardwell and Ristine. Nothing new was elicited in respect to the cause of the disease, we still regarding it an aggravated form of typhoid fever, from using impure water, or from other causes, the nature of which we were unable to ascertain. May 31st, Willie was rapidly sinking; the sustaining treatment ordered evidently affording no relief. At 5 o'clock A. M., June 1st, I again visited my patients, and again renewed my former inquiries in regard to the meat eaten in the family previous to the outbreak of the sickness. I here gained the admission from the ladies of the family that, about the last of April, they had obtained a couple of smoked hams, which were eaten; and that, during Mrs. Lansing's absence from home one day, the little boys had carved off portions of the raw meat and eaten plentifully of it, and the little girls ate some at the same time; and further, that Mrs. Bemis had several times sliced up the raw ham for the table, and it had been eaten, and that a small portion, which remained at my first call, had been thrown away. In from five to ten days, all who had eaten were ill.

Here at once was the solution of the whole difficulty, which I had so earnestly sought previously. Plainly, the disease was trichiniasis. Mr. Lansing had eaten of the meat when well cooked, and had escaped. Mrs. Lansing ate of it very rarely cooked, and had a somewhat mild attack.

I immediately sought Drs. Bardwell and Ristine, and communicated to them the facts I had just learned from the family; to us they were now plain cases of trichiniasis, yet the positive proof was wanting. The same day, Willie L. died, and in the evening, assisted by our leading physicians, I made a limited post-mortem examination, and obtained small portions of the rectus femoris and biceps muscles for microscopic examination.

Upon placing minute particles of these muscles under the glass, the parasites were to be seen in great numbers; the veritable *trichina spiralis.* Many of these were very active, and were readily seen to coil and uncoil, and to exhibit an activity commensurate with the terrible work they had done. I counted 26 trichinæ on one field of the glass; and upon placing a piece of the rectus femoris, $\frac{1}{22}$ of an inch in diameter, under the object glass, 104 trichinæ were counted, which would give nearly 200,000 parasites to the cubic inch. On the 3d of June, Henry B. died, severe pneumonitis having supervened, which no doubt hastened his end. No post-mortem was made. June 8th, Albert Lansing died. In his case anasarca and pneumonitis were prominent symptoms. Not examined after death. The elder Mr. Bemis lingered along until the 15th, complaining of much abdominal pain and great soreness and contraction of the muscles of the extremities.

A post-mortem was held, and the cadaver carefully examined. No lesions of a pathological character were found, the appearance of the viscera being nearly or quite normal. Portions of the muscles, from different parts of the body, were removed for microscopic investigation; also, of the liver, spleen and lungs. The voluntary muscles were swarming with trichinæ; some were found in the lungs and spleen, but none in the heart or liver. On the 17th, Mrs. Bemis died, anasarcous, and with marked pneumonic symptoms. For several days previous to her death, the cough and dyspnœa were so troublesome, while lying down, as to compel her to remain in a half-sitting posture for relief. Her appetite was considerable during the whole course of her sickness, which was not the case with the most of the others. In the treatment I made no experimental efforts, but contented myself with a sustaining course of tonics and alcoholic stimulants, believing that whatever remedies were best calculated to aid nature in her conflict with causes so overwhelming to all the powers of life, were the remedies that common sense and sound medical philosophy indicated. The number of parasites found in the muscles of the bodies examined seems almost incredible, but facts and figures are ugly points to get over, and here amply sustain the statements made. The estimates are fully corroborated by the investigations of very many of the most intelligent and skilful medical gentlemen in Iowa. I also sent a portion of the muscles tested to Dr. E. Ingals, of Rush Medical College, Chicago, and he informs me by letter that the estimates, from careful examinations of the Faculty, correspond very nearly with my own given above. In the last case examined, the number was nearly up to the first; and beside, some encysted worms were found, showing that probably the activity of the parasite begins to subside in from forty to fifty days after the first indications of the malady.

I have now given a concise history of the first authenticated cases of trichiniasis that, to my knowledge, have ever occurred in the western part of the United States. To be sure, none of the trichinous meat eaten was examined, but what is better, the bodies of the victims were scanned, and the parasites were found in multitudes. We farther know that nine persons ate of the raw ham, and in a few days all who had thus eaten, and none others, were taken ill with the same form of disease, differing only in intensity, in proportion to the quantity of raw meat eaten. Five of the nine sufferers have died. The four remaining cases are out of danger, and are regaining their usual state of health.

